# Force and Presence in the World of Medicine

**DOI:** 10.3390/healthcare5030058

**Published:** 2017-09-13

**Authors:** Alan Bleakley

**Affiliations:** Plymouth Peninsula School of Medicine, University of Plymouth, Plymouth PL6 8BT, UK; alan.bleakley@plymouth.ac.uk; Tel.: +44-7713-854138

**Keywords:** force, presence, medical metaphors

## Abstract

Medicine can not only be read with a poetic imagination, but also configured as a poetic practice, moving beyond the instrumental. The poet Wallace Stevens made a distinction between ‘Force’ and ‘Presence’—the former can be read as combative, the latter as pacific. Modern medicine has been shaped historically by the combative metaphor of a ‘war against disease’, turning medicine into a quasi-militaristic culture fond of hierarchy. This is supplemented by the metaphor of the ‘body as machine’, reducing the complex and unpredictable body to a linear, if complicated, apparatus. The two metaphors align medicine with the modern industrial–military complex that is masculine, heroic, and controlling in character. In an era in which medicine is feminising and expected to be patient-centred, collaborative (inter-professional) and transparent to the public as a democratic gesture, the industrial–military metaphor complex should no longer be shaping medicine—yet its influence is still keenly felt, especially in surgery. This continuing dominance of Force over Presence matters because it is a style running counter to the collaborative, team-based medicine needed for high levels of patient safety. Medicine will authentically democratise only as new, pacific shaping metaphors emerge: those of ‘Presence’, such as ‘hospitality’. Hospitals can once again become places of hospitality.

## 1. Wallace Stevens’ Metaphorical Frames

I was beachcombing this morning in the wake of one of the wildest Atlantic swells for many years. The sea was still raging with double overhead waves, each folding over with a crack. As a wave smacked the irritable surface of the pewter grey sea, it rushed up the beach—a white apron—and drained away into the sand to leave a fine mist in which I bathed in gratitude for these gifts of the wild. What struck me was the palpable difference between the force of the incoming tide, capped by those spuming waves unzipping at their crests, and the sudden peace at the high tide strand line as the sand quickly absorbed the spilled contents. Force morphed into presence. A tideline necklace of small gifts—shattered shells and small pieces of polished glass: white, green, and brown—crowned the disappearing trace of the receding wave.

Sea and sky were indivisible, joined by a smoked-oyster haze. The poet Wallace Stevens, in ‘The Man with the Blue Guitar’, says ‘It is the sea that whitens the roof’. The sea reflects white light from the sky back into the underside of the clouds, re-doubling the effort of light. As I turn to the sea on this stormiest of days, where the natural roof is the sky, the sea’s reflection glazes the sky leaden grey, but also adds a tinge of the natural colour of zinc, a bluish-grey. In that moment of beachcombing, and in a reflection of its mass, the sea brought metals—zinc and lead—to the roof of the sky as it redoubled its efforts at a primary demonstration of force. The sky became a literal roof, as a metallic presence. A house of Force was built, within which I made a room of Presence. This house was a hospital made for treating aching bodies. Within it, you could attack your demons as if on a battlefield, or embrace the failings of the body as if in bed with a tender lover.

One of Stevens’ collected proverbs runs: ‘The world is a force not a presence’; however, in the poem ‘St. John and the Back-Ache’ from *The Auroras of Autumn* [1]. Stevens reverses this: ‘The world is presence and not force’. In this poem, Stevens’ character St. John goes on to say that ‘Presence is not mind’, but ‘ … fills the being before the mind can think’, where ‘The effect of the object is beyond the mind’s/extremest pinch and, easily, as in/a sudden color on the sea …’. The mind then is preceded and formed by the senses acutely attuned to the world of objects—the sea’s sudden colour that will surely reflect in the roof of the sky. Pattern recognition, the basis of expertise in medical diagnosis, is grounded in sensory cues and not conceptual clues. The physician Conan Doyle had already taught us this through his character Sherlock Holmes, who notices first and cogitates after. Stevens says that ‘Accuracy of observation is the equivalent of accuracy of thinking.’

Presence is a subtle act of discrimination through close noticing in which the environment educates our attention. Force is the wielding of the blunt object of opinion without the evidence of the senses. In Stevens’ proverb, it is as if Force and Presence are opposed, but I think he would imagine them more as an enantiodromia, where one position can readily turn into the other. A ‘peace-keeping’ presence becomes a force when conflict erupts and returns to presence when conflict passes. Between Force and Presence, at the strand line, things can, in principle, go either way. However, principles are rejected in our ‘post truth’ world in which the opinions of bullies take centre stage and their forcefulness is normalised. Force might be thought of, without stereotyping, as competitive heroic individualism and bullish authority. Presence is the exercise of tolerance shaped by love, compassion and care—that the psychoanalyst Alfred Adler called ‘fellow feeling’ as the exercise of authentic democracy.

I am, then, following Wallace Stevens’ poetic imagination in suggesting that Force and Presence are two big metaphors that frame our thinking and performance. Further, they are not in opposition but liable to enantiodromia, or one turning into the other. Again, Force has engulfed Presence in a post-truth age, where ethically insensitive behaviour is readily normalised. Let us see how these framing metaphors flow down to shape one complex system in our lives—medicine: medical culture, practice, education, and healthcare systems.

St. John can be taken as the ideal of a fcorceful medicine to cure symptoms (‘back-ache’ is pain). Medicine’s martial impulse, the historical emergence, which is considered below, has led to impressive achievements. However, St. John also represents both the arrogance and pieties of medicine. Cure must be balanced by care. In the following section, I articulate—following Foucauldian principles—the historical conditions of possibility for the emergence of Force as a dominant discourse and metaphorical complex in medicine, and ask what this means for contemporary medical practice.

## 2. A Sea Change in Medical Culture

Metaphors are not just poetic devices restricted to literature, but part of the fabric of both everyday discourse and of specialist discourse such as medical practice. Two leading or didactic metaphors have shaped Western medicine: since the 16th century, ‘the body as machine’, and since the 17th century, ‘medicine as war’ [2]. These are metaphors of ‘Force’ rather than ‘Presence’. I will argue that they have forged a medical culture that is no longer fit for purpose: a heroic, masculine, and hierarchical culture that is failing to adapt to an emergent set of healthcare values—patient-centred and team-based, collaborative rather than competitive. Hierarchical, heroic medicine is past its sell-by date. It is time for a medicine in the round that is authentically democratic and embraces the poetic. We need a medicine of hospitality, true to the root of the word ‘hospital’; a medicine of Presence and not Force, shaped by appropriate metaphors. We have outgrown ‘medicine as war’ and ‘the body as machine’. I recognise that it is important to not demonise didactic metaphors. They are not born ‘good’ or ‘bad’ but rather are employed in ways that encompass a moral spectrum. The ‘medicine as war’ and ‘body as machine’ metaphors have driven vital technological and knowledge advances in medicine. However, their transformative energy is spent, indeed there is now a situation of debit.

### 2.1. The Body as Machine

It is easy to see why the metaphor of the ‘body as machine’ has longevity—it is functional, reducing unpredictable complexity to predictable linearity. This is comforting as it reduces uncertainty or ambiguity. Once the body is configured as a machine, it becomes potentially fixable and can be engineered. Of course, this is extremely helpful in many ways, allowing for a range of interventions from targeted drug therapies to neurosurgery. However, bracketing out the difficult bits such as the mind and emotions–or leaving these to the oddballs, the psychiatrists—reduces the complex and abstracted person first to the literal body, second to the complicated linear machine, and third to its component parts—the liver a chemical factory; the heart a pump.

Of course the heart is a pump, but it is also a major attractor in a dynamic, adaptive, and complex system that is the human body embedded in a yet more complex environment. Poetically and psychosomatically, the heart is the locus of love and courage. The mind is surely more complex than its reduction to the matter of the brain modelled in terms of a computer, or generally as electrical engineering, with sick minds a product of faulty wiring. For example, in pain treatment, machine metaphors are legion and often negative, closing off thinking for patients, where ‘locks’, ‘keys’, ‘wires’, ‘circuitry’, and ‘doorways’ are commonly described [3].

The ‘body as machine’ can be traced back to Vesalius’ *Fabrica*, for three centuries the most influential Western anatomy text, where veins, arteries, and nerves are described as hollow tubes ‘like a pipe’. These conduits supposedly worked through pneumatics, where air gives motion to fluid. Nearly a century later, René Descartes (1596–1650) declared that ‘the body is to be regarded as a machine’, explicitly compared the arrangements of the organs in the body with that of ‘automata’ or ‘moving machines’. Giorgio Baglivi (1668–1706), an Italian physician and scientist famous for distinguishing between smooth and striated muscle, deepened this metaphor. Baglivi saw the body as composed of numerous smaller machines: the teeth as scissors, viscera and glands as sieves, the heart and blood vessels as waterworks, and the stomach a flask. Through his sermons, the 18th century Methodist minister John Wesley (1703–1791)—who had studied medicine at Oxford where Thomas Sydenham was a formative influence—described the body as having ‘a thousand tubes and strainers’, any one of which could get obstructed.

Such metaphors still linger, where the lungs are thought of as ‘bellows’, the urinary tract as ‘plumbing’, the liver as a ‘factory’, the heart as ‘ticker’, and various electrochemical mechanisms passing sodium or calcium ions across cell membranes, as ‘pumps’. The rise of neurophysiology, as noted above, brought with it notions of ‘circuits’, ‘switches’, and ‘transistors’, and the brain is finally compared with a computer. In contemporary personalised medicine, based on the genome, health is described as a function of genetic ‘engineering’.

### 2.2. Medicine as War and Illness as A Battle

Susan Sontag (1978) famously railed against the use of martial metaphors such as the ‘war on cancer’, suggesting that we abandon use of metaphor in medicine altogether [4]. This, however, throws the baby out with the bathwater. Metaphors serve various purposes in medicine. The issue is, rather, to shift away from ‘dead’ and stigmatising metaphors to those that vivify.

The war metaphor is so familiar and commonplace in our medical rhetoric that we easily lose sight of its militaristic origins and significance. Notions of ‘fighting’ disease entered Western medicine primarily through the work of Thomas Sydenham in England in the mid-17th century. Prior to that, links between illness and violence metaphors referred to a moral struggle. Where medieval medical texts refer to ‘fighting’ metaphors, these are in the context of fighting sin, so that illness may be metaphorically contextualised as a moral problem. ‘Holy’ and ‘healing’ stem from the same root, where a holy body is a whole body, balanced and well.

Sydenham described medical intervention as if he were vigorously using an assault weapon: ‘I attack the enemy within’, where ‘A murderous array of disease has to be fought against, and the battle is not a battle for the sluggard’. The most famous physician of his day, Sydenham summed up his approach as follows: ‘I steadily investigate the disease, I comprehend its character, and I proceed straight ahead, and in full confidence, towards its annihilation’. Sydenham may have known the poet John Donne’s sermons that included frequent use of ‘illness as violence’ metaphors; and in his writing, such as ‘Devotions Upon Emergent Occasions’, Donne describes his own illness in 1627 as resembling a ‘siege’ and a ‘cannon shot’. Donne thought he was dying from a fever ‘that blows up the heart’, also describing an ‘illness that invades’.

Sydenham’s metaphors did not constitute a dominant discourse at the time. Such a discourse was established in modern medicine two centuries later through Louis Pasteur’s ‘biomilitarism’. While early 19th century doctors often used passive language such as plagues ‘laying’ upon people, Pasteur mobilised an unashamedly active, militaristic language, where diseases ‘attacked’ persons. Pasteur’s description of germ theory displaced a previous language of bodily ‘excess of vital forces’ with a language of invading armies laying siege to the body that becomes a battlefield.

Over a century later, medicine as battle is now a naturalised notion, but once had to be established with militaristic zeal. We know, through computer-assisted corpus linguistic analysis, that violence and war metaphors are common particularly in cancer discourse. Yet research tells us that those cancer patients who view their disease as an ‘enemy’ show higher levels of anxiety and depression, a poorer quality of life, higher levels of pain, and less ability to cope overall than those who represent their illness with a more positive meaning. These findings have been replicated for other conditions such as rheumatoid arthritis. Patients encouraged by their doctors and healthcare workers to ‘fight’ their illnesses report suppressing emotional distress as they put a positive ‘face’ on things in order to not upset both family members and clinicians.

A ‘war against cancer’ was first described in a lead article in the *British Medical Journal* in 1904. This rhetoric was extended to identify the ‘fight against cancer’ as an issue of imperialist domination, where the disease itself was described as ‘darkest Africa’ waiting to be discovered and conquered. Later, cancer cells were identified with Bolsheviks, as ‘anarchic’, threatening the stability of the body. In 1971, Richard Nixon, then President of the United States, delivered a famous speech declaring a war on cancer, where science would ‘conquer’ the disease. At this time, bioscience was replete with martial terms such as ‘killer’ cells and ‘invasive’ species. The gross military metaphor was refined through cellular pathology. As knowledge accumulated about the microorganisms that cause illness, they were described as ‘invading’ the body that produces its own ‘defences’. The body in the early 20th century medicine is configured as a fortress that must be protected from external penetration by germs, or defended from attack. Medicine adopts ‘aggressive’ treatments while the patient is in the line of fire.

Medicine’s hospitality, configured in the hospital, has been forced to adapt to this tough-minded Procrustean bed, so that the hospital itself, and its cells (in particular the operating theatre), become general zones of conflict and specific theatres of war. Violence perpetrated by patients on hospital staff constitutes about three quarters of all workplace assaults and has almost doubled in North America in particular over the past few years [5]. Hospitals can be considered unsafe and unsavoury places to work if harassment and insult by doctors and surgeons aimed at juniors and other healthcare staff is added to the mix of assaults by patients.

Medicine’s saturation in war-making discourse comes to normalise language such as *combating* illness, where *invading* bugs are the *enemy* in the *battlefield* of the patient’s body, that is under *siege* but might be treated with *magic bullets*. Such metaphors extend to nursing culture that is configured as adversarial, where practitioners use phrases such as becoming ‘fatigued by having to do all these battles’, feeling ‘sabotaged’ ‘taking flak’, and working ‘in the trenches’, while their supervisors have ‘deserted the troops’.

The ‘body as machine’ and ‘medicine as war’ metaphors were aligned to produce a powerful frame of reference in the era after the Second World War, as part of a wider cultural ‘military–industrial’ complex. Many doctors had also fought in WWII, and, in North America, subsequently in the Korean and Vietnam wars. The medical gaze became equated with the martial gaze, the battlefield changing through history from the sickroom of the 18th century to the pathologist’s bench of the 19th century, to the imaging room of the 20th century, and to the DNA sequencer’s computer screen of today. Genome sequencing in particular is used to launch ‘precision attacks’ against different types of cancers, where the old military metaphors of combating disease are back in new guise.

The language of war works within its own logic by eradicating its enemies, so it is difficult to spot alternative metaphors. Martial metaphors are inherently bullying—masculine, power-based, paternalistic, and violent or violating. However, patients may tire of this. A patient with colon cancer says that others configuring his illness as a battle ‘was less than palatable’ because: ‘I had already experienced real war in Vietnam and was not anxious to repeat anything closely resembling that.’ It may be exhausting for already exhausted patients to think that they have a battle on their hands, and the notion of victory may be far from the reality. The patient is stigmatised.

Another danger of the dominant militaristic metaphor is that it engages medicine in an arms race, where the dominant fantasy is that all health problems will ultimately be solved with sophisticated technologies as explosive innovations. Further, this metaphor again encourages militaristic organisational structures such as hierarchies with male dominance linked to the aggressive marketing strategies of Big Pharma. Finally, the militaristic metaphor celebrates control and certainty, where much of medicine is in fact about tolerating ambiguity. 

By sticking with, and developing or refining, machine and war metaphors, contemporary medicine operates as if driving a car with the brakes on. How will patients of the future benefit from values, descriptions, and practices that potentially objectify, dehumanise, and stigmatise them and place them in a war zone? Just as medical students learn ‘communication skills’ only to enter practice and interrupt patients on average within 20 seconds of the clinical encounter as a violation of the principle of ‘patient-centredness’, so imagining the body as a machine needing an oil change or a tune up negates the complexity of the illness experience; and perpetuating war metaphors situates patients in a metaphorical landscape not of their choosing.

## 3. Alternative Metaphors to Medicine as War

That didactic or leading metaphors change is established from historical evidence as we have seen. As already noted, it was not always the case that medicine’s interests were described martially. For example, in early modern Europe, cancer might be associated with impurity and rot, or with a corrosive acid, but not with an invading enemy or a hostile presence that has to be killed. The root meaning of cancer is ‘crab’ or ‘crayfish’, and the disease was imagined as surreptitiously moving sideways into surrounding flesh. A commonly used metaphor was that of a rooting (and not even a rotting) plant that was difficult to eradicate and produced seeds, referring to metastasis (from the Greek, meaning ‘a change of position’). Cancer again was typified in terms of a ‘shifting’ presence. The disease was shifty, but not aggressive.

What then are the historical conditions of possibility for didactic metaphors to emerge? As we move into an era of medicine where there are more women than men doctors, where collaborative or democratic inter-professional teamwork is the norm, and where authentic patient-centredness is realised, will militaristic metaphors fade? Yet such metaphors remain stubbornly in place, shaping thinking, and resurfacing, as noted, in the ‘precision attacks’ of personalised medicine. Medicine needs to incorporate new images into its thinking, and essential to this process would be new metaphors around which we can reconstruct both our present and our emerging knowledge. Metaphors can empower patients by making complex medical conditions more understandable and accessible. 

If military metaphors in medicine are historically transient, fraught with contradictions, conceptually weak, and fail to capture the patient’s perspective, it is hard to understand why they have gained such a foothold and maintain traction. Again, it may be that they fit the historically determined cultural form of medicine as quasi-militaristic in its organisation. Further, such metaphors dull the capacity for reflection where their rhetorical style is that of the insistent bully. 

There are emergent alternatives, and they may not be ‘Big Gun’ metaphors but multiple, in combination. In response to the dominant ‘war on cancer’ metaphor, living with cancer has also been described as a chess match, a marathon, a drama, and a dance. Three ‘front runner’ emerging alternatives to ‘medicine as war’ are (i) health as balance and imbalance (homeostasis and dystasis), (ii) medicine as collaborative exploration rather than heroic struggle, and (iii) illness as a journey. 

People suffering in particular from chronic illnesses such as heart disease have characteristically had their identities shaped by the didactic metaphor of ‘illness as a journey’. This metaphor offers rich cross-domain mapping drawing on speed, progress, direction, goals, and pursuit. It is not as aggressive as the martial metaphor and for this reason may be easier to accommodate for patients. Certainly it is the key didactic metaphor in self-help books about illness, now a major genre in literature, and such literature—in the key of New Age therapy-speak—is in danger of trivialising the journey metaphor, or Romanticising it as a new version of the masculine ‘hero’s journey’ with its tired dragonslaying motifs warmed over as fantasy fodder. Such metaphorical frames readily attract and absorb ‘alternative’ therapies, too.

Where heroic, militaristic–industrial complex metaphors can be seen to have shaped dysfunctional healthcare systems, a glaring alternative metaphor is the ‘ecologic’, although this is also prone to being hijacked by New Age interests [6,7]. This metaphor complex includes the pacific and the feminine-collaborative. Concepts from the ecology movement, incorporating systems thinking, can readily be translated across to medicine, including ‘complexity’, ‘dynamicism’, ‘adaptability’, ‘emergent properties’, ‘holism’, ‘integrity’, ‘balance’, ‘natural’, ‘ethical use of limited resources’, ‘quality of life’, ‘diversity’, ‘renewable’, ‘sustainable’, ‘responsibility for future generations’, ‘community’, and ‘conservation’. Thinking with systems challenges the hegemony of ‘body as machine’ metaphors, replacing the linear but complicated (yet fixable) with the complex, unpredictable, and ambiguous.

The use of an ecological and pacific metaphor complex may shift medicine’s primary concern with aggressive treatment to co-ordinated and educated prevention, where population medicine becomes primary; also shifting values away from waste to conservation—from maintaining life at all costs to facing death more realistically. We might also temper false hope in technology-led medicine as saviour and restore ‘small is beautiful’ values and habits of fellow feeling, mutuality, and supportive intervention to communities. Ecological, pacific, and collaborative metaphors work readily together. This shift—from the linear and brutal ‘body as machine’ and ‘medicine as war’ dominant metaphors to the ecologic and systems-based can be seen, in turn, as a shift from ‘Force’ to ‘Presence’.

## 4. Mobilising Wallace Stevens’ Poetics to Energise Medicine

I borrow from Wallace Stevens to frame, and celebrate, medicine and its critical study as poetic and metaphoric practices. Medicine gains by our imagining of it beyond the instrumental to embrace the ethical and aesthetic. The framing of diagnoses and the care of patients have as much to do with form, elegance, beauty, and moral judgement as with function and pragmatism. Stevens’ framing of life’s major tension as the human mind meeting Nature (or Force facing Presence) applies readily to medicine. Presence—natural life (ancient Greek *zoe*)—can be equated with the inevitability of symptom, bodily suffering, and ageing, while Force—cultural life, as the application of the human mind to natural life (ancient Greek *bios*)—is medicine’s response to disease, from planned eradication to management.

Stevens is a particularly good poet to draw upon for re-imagining medicine because his stock in trade is the complex, problematic, and ambiguous. His poetry is at once sensual and highly intellectual, but Stevens wears the intellect lightly, claiming that poetry must ‘resist the intelligence’. It would be easy to see Stevens’ work as a canon of Force, where the critic describes Force as ‘the mind’s violence-from-within’, while Presence is ‘nature’s violence-from-without’ [8]. However, this would be a naive intellectual approach to Stevens’ paradoxical intellectualism, where the poems demand sensory and sensual embrace.

This aesthetic runs beyond the poems themselves to the formats in which they are presented. Stevens was particular about typefaces and the self-display of books. The writer Guy Davenport notes: ‘Stevens’ books, handsomely printed by Alfred Knopf, had an authority and finish, a *presence*’ (my emphasis) [9]. One of Davenport’s own books, *Every Force Evolves a Form* [10], takes its title from the founder of the Shakers, Mother Ann Lee. The Shaker aesthetic is a precursor to Minimalism, where, like the work of the iconic Shaker broom, the mess is brushed away to leave a clean, sanitised space. Neither messy medicine, meeting ambiguity and uncertainty at every turn, nor Wallace Stevens’ complex poetry can be said to outwardly display a Minimalist aesthetic, but both Stevens’ poetry and medicine are practices built with Minimalist scaffolding. Medical education stresses that the patient must be compressed to the ‘case’, and the ‘case’ further presented as a collapsed narrative, framing symptom as figure against ground. Stevens’ poetry has a similar skeletal structure made evident through the X-ray of exegesis. For example, Austin Allen shows how Stevens’ famous elliptical poem ‘The Emperor of Ice-Cream’ brings together two scenes in one dwelling: ice cream is being whipped up in the kitchen as part of a wake meal prepared for mourners of the woman who lived in the house, recently dead, while her corpse is being prepared to be laid out [11]. Force—culture’s mindful resistance to Nature—is exhausted; Presence rules. Yet regal Force re-appears as short-lived, the Emperor as whipped ice cream, nature briefly transformed.

All such readings of Wallace Stevens’ work are conditional. Despite Harold Bloom’s confidence in his analysis of the poem ‘St. John and the Back-Ache’ [8], the same poem 30 decades on from Bloom is described by Eleanor Cook (2007) as a ‘Remarkable debate-poem on the question of pain, still in need of good exegesis’ [12].

## 5. Conclusions

Modern medicine has been shaped historically by an ‘industrial–military’ complex of metaphors—‘body as machine’ and ‘war against disease’—that I read, borrowing Wallace Stevens’ metaphors, as the dominance of ‘Force’ over ‘Presence’. Contemporary medicine demands collaborative, patient-centred, inter-professional teamwork. This promises a medicine that is both feminised and democratised, guided by an enlightened medical education. However, for this progressive medicine to gain traction, new shaping metaphors must emerge. Likely candidates are ecological metaphors such as holism, and medicine enacted as authentic hospitality. Stevens’ ‘Presence’ described at the beginning of the essay has morphed into this author’s wider interpretation to embrace democracy, feminisation, collaboration, and tenderness. This creative drift is necessary if we are to make sense of how the metaphor of Presence is translated into contemporary clinical teamwork as patient-centred and inter-professional practices.
